# The importance of the integration of community engagement with biomedical informatics when assessing social determinants of health

**DOI:** 10.1017/cts.2025.15

**Published:** 2025-03-19

**Authors:** Linda B. Cottler

**Affiliations:** Department of Epidemiology, University of Florida, Gainesville, USA

The importance of community engagement (CE) has increased in recent years in large part due to the launch of the NIH Clinical and Translational Science Award (CTSA) program. For the first time, sites were required to focus on CE, and as a result, they partnered with agency directors, faith-based leaders, providers, and the community at large. Consequently, social determinants of health (SDOH) were noticed, and addressed systematically, from educational attainment, employment, access to health services, housing, zipcode, food insecurity, physical and social environment, transportation, and others. CE programs listened to the community to learn how addressing SDOHs would help tailor interventions and alter policies to improve health equity – one of the goals of CE.

Over the years, the science of CE research has matured which has led to scientists updating the “principles of community engagement.” The 1^st^ Edition was published in 1997 [1], the 2^nd^ 14 years later in 2011 [2]. These versions described 9 principles; the 3^rd^ Edition, published in 2025, added a 10^th^ – “trustworthiness.” The new definition of CE highlights the need to communicate multidirectionally, to be solution-oriented, and to focus on outcomes that matter to the community [3]. There is also a discussion in the 3^rd^ Edition on the critical need to align and integrate CE goals with those of the field of biomedical informatics to better promote equity and reduce disparities. An informatics approach could elevate the success of CE programs and goals. To achieve this integration/alignment, the two disciplines need a better understanding of each other’s areas, which requires bidirectional education. Input from a CE scientist into the biomedical informatician’s world enhances biomedical informatics. Input from the biomedical informatician’s perspective into CE would bolster CE research.

As you read the papers in this supplement, consider what more we need to integrate the CE and biomedical informatics disciplines. What resources could be provided for CE to achieve parity with the resources that are available for biomedical informatics? How can our institutions make these efforts durable? Long-lasting? CE investigators, who focus their work on the health of populations in their communities, have a big goal (population health) with a small budget. The bar for CE efforts to show impact is always set very high. How could biomedical informatic strategies facilitate this goal?

A call to action was posed by the Rowland et al. paper [4] regarding Digital Health Equity. The authors rightly stated that minoritized groups have less access to telemedicine than non-minoritized groups, such as Non-Hispanic Whites. Using a health equity lens to digital health through education and training, the authors utilized broadband access and digital literacy tools. They recommended that CTSAs provide their own resources for pilot projects to test the utility of digital tools, mentioning Patient-Centered Outcome Research Institute and WHO guidelines. These guidelines are not universally available to all communities and cannot turn Academic Health Centers into trustworthy partners. The project’s use of videoconferencing and apps was described as an informatics best practice for CE along with the reliance on Focus Groups and Community Advisory Boards (CABs). To strengthen this framework and optimize the outcome of this effort, a biomedical informatician could have been included.

Improvements in assessing SDOH were described in the Heinert et al. [5] paper which included a CHW model referred to as a Citizen Scientist approach. A public-school course was developed where students interviewed adults in their community about their health needs; those data were compared to those collected in the academic hospital. Community members reported more discrimination, more food insecurity, and more barriers to care than participants interviewed by their counterparts. The authors concluded that hospitals should consider partnerships with local high school students to find a more “representative” sample. As an epidemiologist, I worry about social desirability bias when people (in this case, students) collect personal data from relatives and friends. Could it over or underestimate the factor of interest? Regardless, students learned about primary data collection, learned about social determinants, planned health fairs, and thought about policies that could be developed to mitigate these factors. This implementation project, launched during COVID-19, was successful in showing how high schoolers both learned about SDOH and developed pragmatic solutions for their community. Biomedical informatics strategies were never mentioned, though it is easy to imagine how they could have been successfully included.

Another effort conducted during COVID was one by Lee et al. [6] focused on the LGBTQIA2+ community. While surveys and assessments ask about gender as an SDOH, many patients and participants question the need for the provider or research team to know this information if there is no improvement in services as a result. LGBTQIA2+ persons are underrepresented in research because of the data being both discoverable and non-discoverable, or when people are unwilling to participate when needs are not met by the clinical trial team. While 85 patients signed up through a student-led Rainbow Clinic, only 49 were seen due to cancelations or “no shows.” The authors suggested a few SDOHs (food insecurity, unemployment, being uninsured or underinsured, poor mental health conditions, and being unhoused) might have contributed to low participation. While there were no comparison data, it was perceived that people in this study felt comfortable coming to a particular clinic where they might avoid being discriminated against. Besides showing the importance of being a trustworthy partner, this study highlighted how digital health might have assisted in more successful data collection.

The Huang et al. [7] paper identified incarceration status – an important SDOH – using language models in the Emergency Department setting using EHR. While the authors detailed the importance of this history for tailored interventions and interventions that focus on barriers to housing, primary care services, and others, digital health information was never mentioned for providing referrals. It was also unclear if someone with lived experience was included on their team. My lab has found one of the most difficult Human Resource logistics is hiring someone who has been incarcerated. A biomedical informatician’s input was needed due to the potential for an ethical problem if this history was inaccurately estimated. The team developed a Natural Language Processing model (the Clinical Longformer) to identify recent incarceration history “reliably and accurately,” to facilitate health services research and referrals. Misuse and the unethical use of the tool was addressed. In the past, ED providers might have been influenced by visual proxy signs that might hint at a criminal background such as “needle tracks.” Community voice was sorely needed here to help educate providers on how to take a history of incarceration from the patient where discrimination could be reduced or eliminated rather than relying on artificial intelligence.

The Vargas et al. [8] paper detailed a COVID-19 issue in their study of 212 people who self-reported having Vietnamese ethnicity. Unfortunately, analyses of SDOH were not representative because of the large number of people who preferred not to answer pertinent questions. CE involvement with a Community Health Worker might have helped mitigate this issue if the authors had invited them to review the survey questions. Only 30% of the population reported being willing to participate in COVID-19 Clinical Trials. It would have been important to see how this compared to people of other ethnicities. This paper highlighted the need to include people of Asian ethnicity and the importance of translating surveys. Without this, there is less opportunity to evaluate the effect of SDOH across minoritized groups. The high trust in pharmaceutical and university hospital research they found contradicts the Liu et al. [9] study of ∼ 17,500 community members from 6 CTSAs showing that Asian Americans were the least willing of all race/ethnic groups to be willing to participate in 8 different types of research, but as likely as others to trust researchers [9]. It is unclear how a biomedical informatics approach could have helped.

Crowe et al. [10] addressed SDOH related to COVID-19 vaccine disparities through a university public health partnership. Their “toolkit” explored the Translational Science Benefits Model and how it could be used to evaluate the effectiveness of a partnership model. The team tracked demographics, events, food distribution, and vaccine hesitancy; however, no data were reported on the impact of the events other than overall numbers and unique funding offered by the local agencies – something needed in other communities. Here as well, the informatics approach would have failed if data were not properly collected.

The McKinney et al. [11] paper evaluated the “impact” of SDOH on cognitive development and individual differences in IQ among persons with syndromic intellectual developmental disabilities. The authors identified areas for future research to potentially shape community-level interventions that improve health for people living with IDDs and urged researchers to consider including the assessment of SDOH in all research. CE scientists would agree with this important suggestion, as well as the inclusion of family as valuable, authentic members of the team, even perhaps as a co-author. Certainly, their team science approach would benefit from the recommendations of a biomedical informatician.

The Ritchie et al. [12] article on enrollment of Underrepresented Populations (URPs) in a diabetes clinical trial consisted of using phone and videoconferencing. The authors found that allowing remote access was associated with a 25% enrollment rate vs a 10% enrollment rate through an in-person strategy and that increased enrollment for minoritized persons and persons with overweight and obesity was also higher when remote (digital) options were offered. They attributed this to less perceived stigma and discrimination because remote options “made people feel more comfortable.” It could also have been important to “meet people where they are” by conducting outreach in rural areas. In addition, as the authors note, the data were from only one site and one health system. It is unclear how a biomedical informatics approach could have helped to determine an additional subset of the population or data collection itself.

The final study in the group of CE papers was from Kumpf et al. [13]. This case study offered a model of community-driven partnership in a collaborative effort with a CTSA that was centered around health fairs that addressed health education, screenings, vaccinations, and other resources. Their 2.5-year project included a Health Needs Assessment over Zoom where Community Based Organization (CBO) representatives shared their ideas of what their constituent’s needs were. The CBO representatives along with others developed an action plan. Attendees participated in a post-event survey about their satisfaction, interventions, and even what giveaways they should offer. The case study provided an example of how needs can be addressed with direct interventional referrals. Unfortunately, no demographic data were assessed which limited their ability to evaluate impact. However, the rules of CE were firmly established. Even though this described a robust CE outreach, it would not have been easy for an informatics lens to be applied.

Several things are important to note from this group of papers in this supplement. The field of CE involves people with strong creativity, who are perspicacious and pride themselves on being solution-oriented. They have their fingers on the pulse of their community which makes them excellent partners with the community and with biomedical informaticians. Scientists involved in Community Engaged Research listen to and learn directly from the community, so they know the issues that are a priority to them. This made CTSAs transformative. Now, they must take this transformation to the next level and involve the entire community in all stages of translational science, from the bench to the community. In this way, the need transforms a person’s access to a health provider, a particular health system, or even if the person has a medical record.

Scientists are learning to integrate self-report community accessed data with clinical data (if there is a record) to understand SDOH from a community of people served vs a community not served and best practices to obtain data from the person themself. This would be an excellent return of value to communities. The needs and concerns identified could be shared with academic institutions and Community Advisory Boards alike to make these areas priority for pilot studies, policies, and interventions.

A program such as HealthStreet at the University of Florida is one such example that meets people where they are. CHWs assess needs, and refer people based on those needs to medical and social services and opportunities to participate in UF research [3]. Outcomes and outputs are tracked. This emphasis can help discover best approaches for enrolling, but there is a strong need for help from the informatics field as CE scientists are exploring hypothesis-driven pragmatic trials focused on how to better recruit participants from the entire community, how to use prediction models that utilize a person’s trust score to achieve high retention, and how to address SDOH to suggest relevant social prescribing efforts once needs are discovered.

This is a great time for a shared perspective on the integration of AI/Machine Learning/Digital Health with the science of community engagement (CE). It was no accident that many of the papers in this supplement focused on COVID-19-era principles. COVID-19 was a wakeup call for institutions to not turn their backs on the community they had been serving. Institutions realized that they needed their CE programs which led a group of CE scientists to call on funders and institutions to take action [14,15]. This call to action is still needed [16]. Just as biomedical informaticians are the experts in algorithms, statistical ideas, and models, community-engaged scientists are the experts in connecting with the community and assessing SDOH. Both of our fields can help select the subgroups needed, design studies, and interpret the findings. The highest level of integration of both fields will ultimately improve the health of our entire community and supplements such as this can help pave the way.

## References

[1] Centers for Disease Control and Prevention. Principles of Community Engagement (1st edition). Atlanta (GA): CDC/ATSDR Committee on Community Engagement: 1997.

[2] Clinical and Translational Science Awards Consortium, Community Engagement Key Function Committee, Task Force on the Principles of Community Engagement, Agency for Toxic Substances and Disease Registry, Centers for disease control and prevention (U.S.). Principles of Community Engagement (2nd Edition). Atlanta (GA): NIH Publication No. 11-7782: 2011, pp 1–193. https://stacks.cdc.gov/view/cdc/11699

[3] Centers for Disease Control and Prevention. Principles of Community Engagement (3rd Edition). Atlanta (GA): 2024. Cohn E and Berman L (Eds). https://hsc.unm.edu/population-health/_documents/principles-of-community-engagement_3rd-edition.pdf Accessed March11, 2025.

[4] Rowland S , Brewer LC , Rosas LG. Digital health equity- a call to action. J Clin Transl Sci. 2024;8(1):e145. doi: 10.1017/cts.2024.564. 39478785 PMC11523008

[5] Heinert SW , Ciezak J , Clifford J , et al. Engaging youth as citizen scientists to determine health needs of New Brunswick adults. J Clin Transl Sci. 2023;7(1):e253,1–6. doi: 10.1017/cts.2023.674. 38380393 PMC10877509

[6] Lee GA , Fritter J , Shihabuddin CD. Increasing safe clinical spaces and the efforts of clinical research for uninsured and underinsured LGBTQIA2+ patients: A case study of the rainbow clinic- a student-run free LGBTQIA2+ clinic. J Clin Transl Sci. 2023;7(e218):1–5. doi: 10.1017/cts.2023.641. PMC10643935 38028348

[7] Huang T , Socrates V , Gilson A , et al. Identifying incarceration status in the electronic health record using large language models in emergency department settings. J Clin Transl Sci. 2024;10(doi):10.1017/cts.2024.496. PMC10966832 38544748

[8] Vargas S , Siddiqi S , King B , et al. Vietnamese Americans’ level of trust in sources of information and willingness to participate in COVID-19 clinical trials. J Clin Transl Sci. 2024;8(1):e88. doi: 10.1017/cts.2024.495. 38784109 PMC11112424

[9] Liu Y , Elliott A , Strelnick H , Aguilar-Gaxiola S , Cottler LB. Asian Americans are less willing than other racial groups to participate in health research. JCTS 2019;3(2-3):90–96. doi: 10.1017/cts.2019.372 PMID: 31660231; PMCID: PMC6802418. 31660231 PMC6802418

[10] Crowe S , Adeoye-Olatunde Kimiecik C , etal OA. Social determinants of health-based strategies to address vaccination disparities through a university-public health partnership. J Clin Transl Sci. 2024;8(e66):1–6. doi: 10.1017/cts.2024.502. PMC11058580 38690220

[11] McKinney WS , Williford DN , Abbeduto L , Schmitt LM. The impact of social-environmental factors on IQ in syndromic intellectual developmental disabilities. J Clin Transl Sci. 2024;8(e59):1–15. doi: 10.1017/cts.2024.510. PMC11036438 38655457

[12] Ritchie ND , Turk MT , Summers Holtrop J , josh Durfee M , Dickinson LM , Kaufmann PG. A virtual recruitment protocol promotes enrollment of underrepresented groups in a diabetes prevention trial. J Clin Transl Sci. 2024;8(e26):1–5. doi: 10.1017/cts.2024.11. PMC10879998 38384920

[13] Kumpf E , Thamilselvan V , Wang E et al. Community-driven partnerships with CEnR Teams bring resources and reliable information to Baltimore residents. J Clin Transl Sci. 2024;8(1):e205. doi: 10.1017/cts.2024.606. 39655015 PMC11626593

[14] Grumbach K , Cottler LB , Brown J , et al. It should not require a pandemic to make community engagement in research leadership essential, not optional. J Clin Transl Sci. 2021;5(1):e95. doi: 10.1017/cts.2021.8. 34192052 PMC8134899

[15] Eder MM , Millay TA , Cottler LB. A compendium of community engagement responses to the COVID-19 pandemic. J Clin Transl Sci. 2021;5(1):e133. doi: 10.1017/cts.2021.800. 34367677 PMC8326670

[16] Meissner P , Cottler LB , Eder MM , Michener JL. Engagement science: The core of dissemination, implementation, and translational research science. J Clin Transl Sci. 2020;4(3):216–218. doi: 10.1017/cts.2020.8. 32695491 PMC7348030

